# Arts of electrical impedance tomographic sensing

**DOI:** 10.1098/rsta.2015.0329

**Published:** 2016-06-28

**Authors:** Mi Wang, Qiang Wang, Bishal Karki

**Affiliations:** School of Chemical and Process Engineering, University of Leeds, Leeds, West Yorkshire LS2 9JT, UK

**Keywords:** electrical impedance tomography, sensitivity, resolution matrix, regional imaging with limited measurements

## Abstract

This paper reviews governing theorems in electrical impedance sensing for analysing the relationships of boundary voltages obtained from different sensing strategies. It reports that both the boundary voltage values and the associated sensitivity matrix of an alternative sensing strategy can be derived from a set of full independent measurements and sensitivity matrix obtained from other sensing strategy. A new sensing method for regional imaging with limited measurements is reported. It also proves that the sensitivity coefficient back-projection algorithm does not always work for all sensing strategies, unless the diagonal elements of the transformed matrix, A^T^A, have significant values and can be approximate to a diagonal matrix. Imaging capabilities of few sensing strategies were verified with static set-ups, which suggest the adjacent electrode pair sensing strategy displays better performance compared with the diametrically opposite protocol, with both the back-projection and multi-step image reconstruction methods. An application of electrical impedance tomography for sensing gas in water two-phase flows is demonstrated.

This article is part of the themed issue ‘Supersensing through industrial process tomography’.

## 1. Introduction

Electrical tomography, including the measurement based on electrical property, e.g. electrical impedance tomography (EIT) or dielectric property, e.g. electrical capacitance tomography (ECT), has been developed since the late 1980s to provide alternative, low-cost solution for clinical, geophysical and industrial process applications [1]. A number of sensing strategies for EIT have been developed in previous years. The most common choices were four-electrodes measurement, e.g. the adjacent electrode pair protocol [2] and diametrically opposite protocol [3] in EIT, or three-electrodes measurement, e.g. the metal wall protocol [4] in EIT and that used in most of ECT systems [5,6]. However, there is as yet no general agreement for the ‘best’ sensing strategy in this area [7]. Based on the reciprocity and sensitivity theorem proved by Geselowiz [8] and Lehr [9] and the assumption cited for sensitivity coefficient back-projection (SBP) approximation [10], the principles of reciprocity and independency and also imaging capability in EIT are discussed in this paper.

## 2. Governing theorems

### (a) Lead theorem and reciprocity theorem

The lead theorem was derived from the divergence theorem (equation (2.1)) by Geselowitz [8] and Lehr [9] for the impedance plethysmography.
2.1
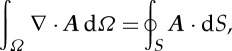
where *Ω* is a region bounded by a closed surface *S*. ***A*** is a vector function of the position.

The mutual impedance *Z* of a four-electrode system with a known conductivity distribution can be derived by substituting *ψ****J***_*ϕ*_ or *ϕ****J***_*ψ*_ into the divergence theorem (equation (2.1)) and then substituting ***J***_*ϕ*_=−*σ*_*ϕ*_∇*ϕ* or ***J***_*ψ*_=−*σ*_*ψ*_∇*ψ* into the left side of the reformed expression under the condition of no internal current source [4], where *ψ* and *ϕ* are potential distributions in response to the presence of currents *I*_*ψ*_ and *I*_*ϕ*_ at two ports (*A*–*B* and *C*–*D*), respectively.
2.2
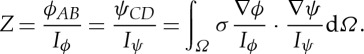
The mutual impedance change Δ*Z* owing to a change of internal conductivity Δ*σ* for a four-electrode system, derived by Geselowits and Lehr and later linearized by Murai & Kagawa [11], is given as equations (2.3) and (2.4), respectively. Equation (2.4) ignores the high-order term and provides a linear relationship with an assumption of Δ*σ*≪*σ*. Equations (2.2) and (2.3) are also called the reciprocity theorem.
2.3
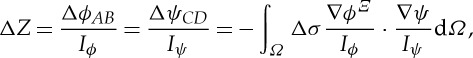
where *ϕ*^*Ξ*^ is the potential change caused by the conductivity after change *σ*^*Ξ*^=*σ*+Δ*σ*
2.4
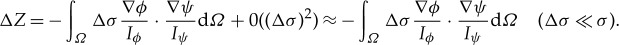


### (b) Sensitivity theorem

Supposing that the conductivity distribution is composed of *w* small uniform ‘patches’ or pixels, then equations (2.2) and (2.4) can be expressed as equations (2.5) and (2.6) and the sensitivity coefficient *s* for each discrete pixel is given by equation (2.7) [11–13].
2.5
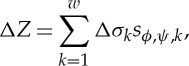

2.6
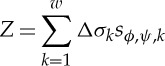

2.7
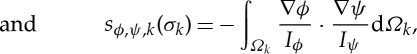
where *Ω*_*k*_ stands for a discrete two-dimensional area at location *k*; *σ*_*k*_ and Δ*σ*_*k*_ denote the conductivity and the change of conductivity *Ω*_*k*_, respectively.

**Figure 1.**
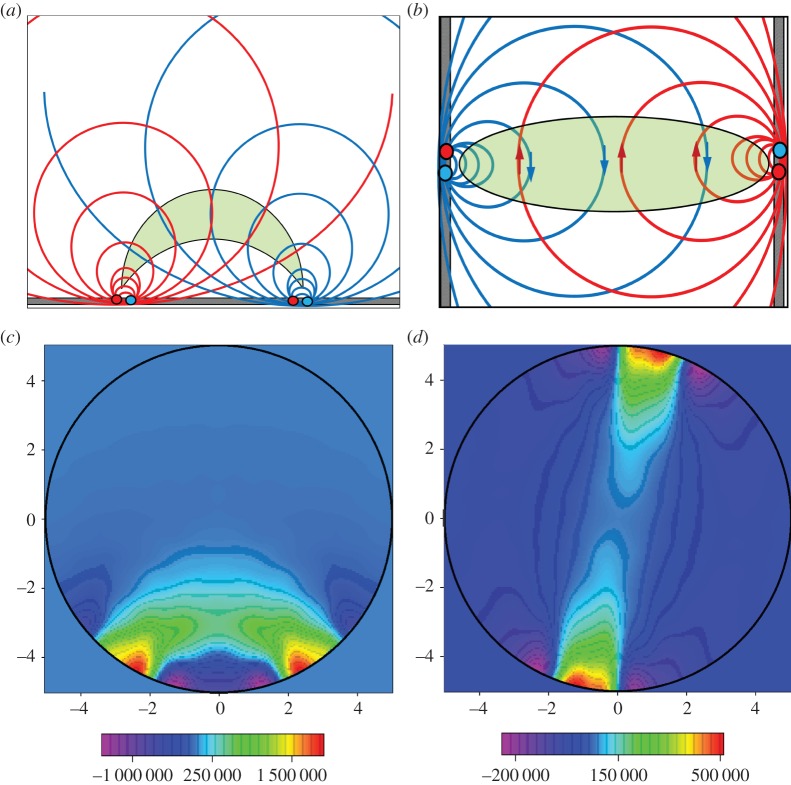
Estimation of EIT sensing bands with the graphic analogue method and numerical simulation method. Panels (*a*,*b*) are from the graphic method where the red and blue spots denote the current flowing in and out electrodes by applying a current; the red and blue curves are the equal-current density streams which illustrates the electric field distribution generated by relevant electrode dipoles. Panels (*c*) and (*d*) are simulated with FEM for a two-dimensional disc-shaped domain, where the fields are existed by a current on electrode pair 3–4 and 7–6, 4–5 and 12–13, respectively.

Equation (2.7) indicates that the sensitivity at a defined location is a vector scalar production of two electrical fields generated by current excitations on source and measurement electrode pairs (equation (2.7)). In other words, the sensitivity will be maximized at a location when two electric fields are mostly in parallel. With the orthogonal principle between the electrical potential and current density (electrical field intensity) of two electric dipoles, figure 1 illustrates the sensing ‘bands’ produced by two pairs of electrodes positioned on a non-conductive plate and an opposite position, respectively. The graphic method (figure 1*a*,*b*) is a simple way to initially estimate the sensitive region from a setting up of electrode allocation for process vessels having different geometry, where the sensing ‘bands’ are highlighted in green. However, no sharp boundary of bands exists in an actual sensitivity distribution owing to the use of low-frequency electromagnetic excitation. In practice, the sensitivity coefficients are normally computed with either analytical or numerical methods. Figure 1*c*,*d* demonstrates the sensitivity distributions simulated with finite-element modelling method (FEM) for a 16-electrode sensing array, where figure 1*c* is generated by excitation and measurement electrode pairs 3–4 and 7–6 and figure 1*d* is from electrode pair 4–5 and 12–13. For a good sensing strategy, it is necessary the coverage scanning and cross-band scanning can be made over the domain of interests.

### (c) Inverse solution

For a linear equation (2.8), the minimization function (2.10) can be obtained from minimizing equation (2.9).
2.8
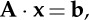

2.9
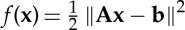

2.10
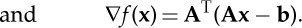
Let ∇*f* = 0, then,
2.11
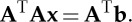
If an inverse matrix of **A**^T^**A** exists, then the solution can be made as equation (2.12)
2.12
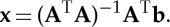
It is known the inverse matrix of **A**^T^**A** is not derivable owing to the ill condition of sensitivity matrix **A** in EIT. Many indirect methods were developed to solve the linear equation in the past. Typically, they were reported as the back-project method [14] and the Newton one-step reconstruction (NOSER) single step method [15]. However, it should be pointed out the solution for equation 2.12, if any exists, only satisfies the change of conductivity Δ*σ*≪*σ*. The closest solution may only be obtained from multi-step approach [16].

#### (i) Back-projection methods

**Figure 2.**
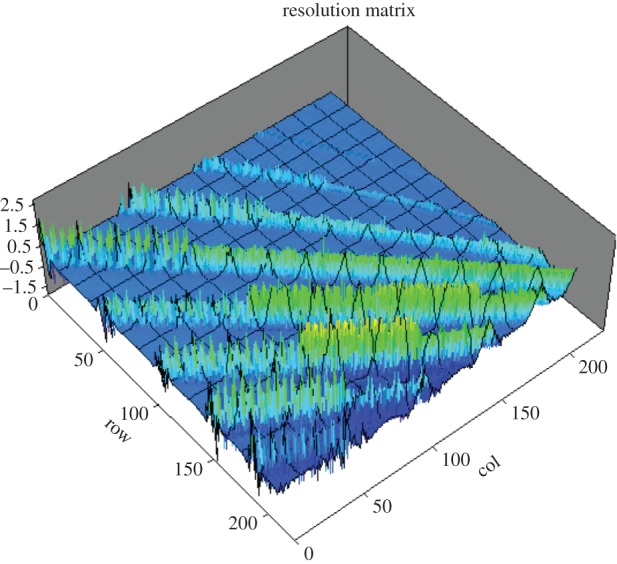
Resolution matrix for a 16-electrode sensing array using the adjacent electrode pair sensing strategy with a two-dimensional mesh with 224 pixels.

Wang [10] indicated that the sensitivity coefficient back-projection method (SBP) is actually based on an assumption of that the diagonal elements of the transformed matrix, **A**^T^**A**, have the most significant values and the matrix can be approximate to a diagonal matrix. For a particular example of adjacent sensing strategy, the solution to equation (2.10)) with ∇*f*=0 is firstly normalized to equation (2.13), then approximate to equation (2.14) if the ***τ*A**^T^**A** can be approximated as an identity matrix.
2.13
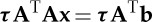
and
2.14
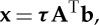
where ***τ*** is the inversed matrix for the approximated diagonal matrix of **A**^T^**A**(**A**=[*a*_*ij*_]), in which its diagonal parameters satisfy
2.15
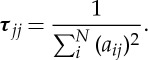
The transformed matrix in equation (2.13) or also called the resolution matrix [17], **R**=***τ*A**^T^**A**, presents the pixels' correlation in EIT, which is the same as that presented by the Hessian matrix in Newton–Raphson (NR) method [16]. If none of the pixels is correlated, then the Hessian matrix would be a diagonal matrix [18]. This feature of diagonal domination is commonly interpreted as evidence for high-quality inversion [17]. However, cross-correlations are among all pixels in EIT, which produce a skew form of an optimized matrix, in which the diagonal elements may have most significant values. Figure 2 demonstrates the significance of diagonal components in the resolution matrix from the adjacent electrode pair sensing strategy, which are modelled using FEM with a mesh having 224 pixels for a 16-electrode sensing array. Equation (2.14) with the existence of the diagonal matrix approximation provides the mathematical principle for the SBP algorithm.

#### (ii) Multi-step methods

The linear approximation, based on ignoring the high-order terms with respect to Δ*σ*≪*σ* (see equation (2.4)), enables the use of iterative techniques to solve the linear equation (equation (2.11)) with a pre-calculated sensitivity matrix based on an estimated initial conductivity distribution, e.g. a homogeneous distribution. However, in most of cases, multi-steps of the linear solution process have to be applied in order to achieve better image quality, where the sensitivity matrix should be updated with the change of conductivity distribution obtained from the previous step. Its performance is strongly associated with the methods and the convergent factors used in iterative minimization process as given by equation (2.16). The typical examples are the use of NR optimization [16], Tikhonov regularization method [19,20] with the techniques of selection of regularization factors such as the singular value decomposition method and Akaike's information criterion [21] and the Marquardt method [22].

The error function is
2.16
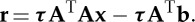


#### (iii) One-step methods

One-step method refers the solution from the iterations at the end of the first step of a multi-step method. It is the fact that the result from the first-step solution normally provides the most contributive convergence if the regularization factors are properly selected. The quality of resultant images is generally much better than those came from back-projection approximation as results from NOSER method [15] and the SCG method [10]. It also runs at a much fast speed than that of multi-step method owing to the use of the pre-calculated sensitivity matrix. An iterative solution can be obtained in a kind of the Landweber iteration method as expressed by equation (2.17) [23].
2.17
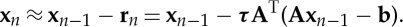
The selection of ***τ*** for the SBP has been suggested by equation (2.15). An example for the use of Landweber's method with selection of *τ* for capacitance tomography was reported by Liu *et al.* [24] and also many other recent publications. For a general application, the selection of *τ* can be referred from the Landweber method [23].

## 3. Sensing strategy

### (a) Electrode–electrolyte interface

**Figure 3.**
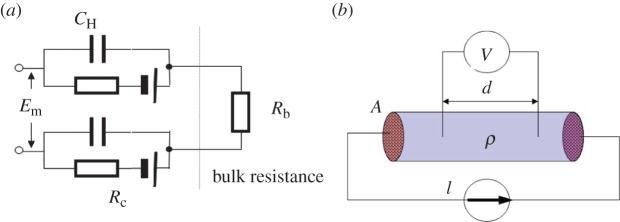
(*a*) The electrode–electrolyte interface; (*b*) four-electrode method. (Online version in colour.)

A metal electrode immersed in an electrolyte is polarized when its potential is different from its open-circuit potential [25]. The electrode used in EIT is a transducer that converts the electronic current in a wire to an ionic current in an electrolyte. The behaviour at the electrode–electrolyte interface is a predominantly electrochemical reaction [26]. The electrode–electrolyte interface can be described by a simple circuit as shown in figure 3*a*, where *R*_C_ denotes the charge transfer resistance, *C*_H_ represents the double layer capacitance, *E*_m_ is the over potential difference and *R*_b_ is the bulk resistance of a process. All of these three major representatives are function of the ionic concentration of the electrolyte, surface area and condition of electrode, and also current density over the interface. The conventional and effective way to avoid error caused by the interface in the measurement of bulk resistance is to use a four-electrode method as shown in figure 3*b*. In principle, the voltage measurement, *V* , should not be a function of the two interfaces' electrochemical characteristics because the current, *I*, remains constant flowing through any cross section of the cylindrical object. The four-electrode method is also widely used in EIT [1], typically, as it is used in adjacent electrode pair sensing strategy.

### (b) Sensing strategies

**Figure 4.**
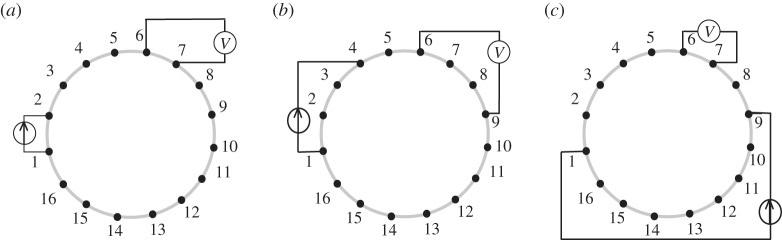
Different sensing protocols: (*a*) adjacent sensing strategy, (*b*) PI/2 sensing strategy, (*c*) opposite sensing strategy.

In EIT, the boundary conditions required to determine the electrical impedance distribution in a domain of interest comprise the injected currents via electrodes and subsequently measured voltages, also via boundary electrodes. A number of excitation and measurement methods or sensing strategies have been reported since the late 1980s [1,27]. Figure 4 shows three different sensing strategies under the review, which are adjacent electrode pair strategy [2] (figure 4*a*), an alternative sensing strategy (figure 4*b*), which is named as PI/2 protocol owing to a *π*/2 radian between its nearest measurement electrode pairs [28], and the diametrically opposite sensing strategy [3] (figure 4*c*). Applying the reciprocity theorem or leads theorem as given by equations (2.2) and (2.3) and also in the use of four-electrode method, the number of independent measurements from these strategies (ignoring the measurements from current drive electrodes) is 104, 72 and 96, respectively.

### (c) Linear relationships

Taking the example of the measurement and excitation positions in the PI/2 sensing strategy (figure 4*b*), its boundary voltages can be derived from the adjacent electrode pair sensing strategy based on the theorem of circuitry. Firstly, we have
3.1
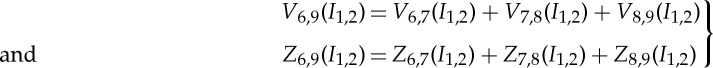
where *V*_6,9_(*I*_1,2_) denotes the voltage measured from the electrode 6 and 9 when a current presented between the electrode 1 and 2, *Z*_6,9_(*I*_1,2_) represents the mutual impedance, *V*_6,9_/*I*_1,2_.

According to the reciprocity theorem, the mutual impedance can be expressed as
3.2
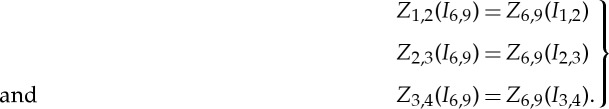
Following the principles of equations (2.2) and (2.4), the mutual impedance of the circuitry in figure 1*b* can be presented as
3.3
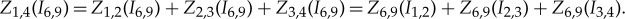
Substituting equation (3.1) into equation (3.3) with the reciprocity given by equation (2.4), the mutual impedance of the circuitry in figure 4*b* can be described using a set of the mutual impedances derived from the circuitry in figure 4*a*
3.4
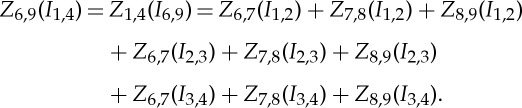
Further, the boundary voltages in figure 4*b* can be expressed with a set of boundary voltages of the circuitry in figure 1*a*.
3.5
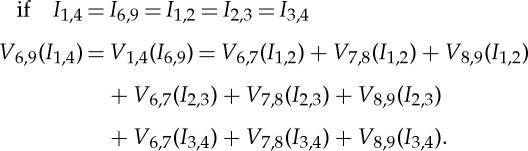
A general form of the relationship between an alternative four-electrode sensing strategy and the adjacent sensing strategy in EIT can be expressed as equation (3.6), which derives a mutual impedance or a boundary voltage for an alternative four-electrode sensing strategy from a set of mutual impedances of the adjacent electrode pair strategy.
3.6
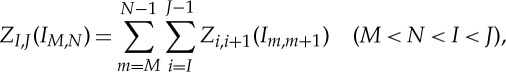
where *M* and *N* denote the current drive electrode pair, *I* and *J* denotes the measurement electrode pair of the alternative four-electrode sensing strategy.

Because a linear approximation has been adopted in the calculation of sensitivity matrix [8,9,11] and a linear relationship of the boundary voltages exists for different four-electrode sensing strategies (equation (3.6)), the sensitivity matrix of an alternative sensing strategy can also be derived from the sensitivity matrix obtained from a complete set of independent measurements of other sensing strategy. Equation (3.7) gives the derivation from the sensitivity matrix of the adjacent electrode strategy.
3.7
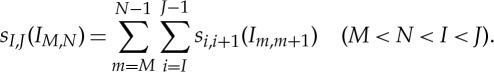


### (d) Regional imaging based on reduced measurements

**Figure 5.**
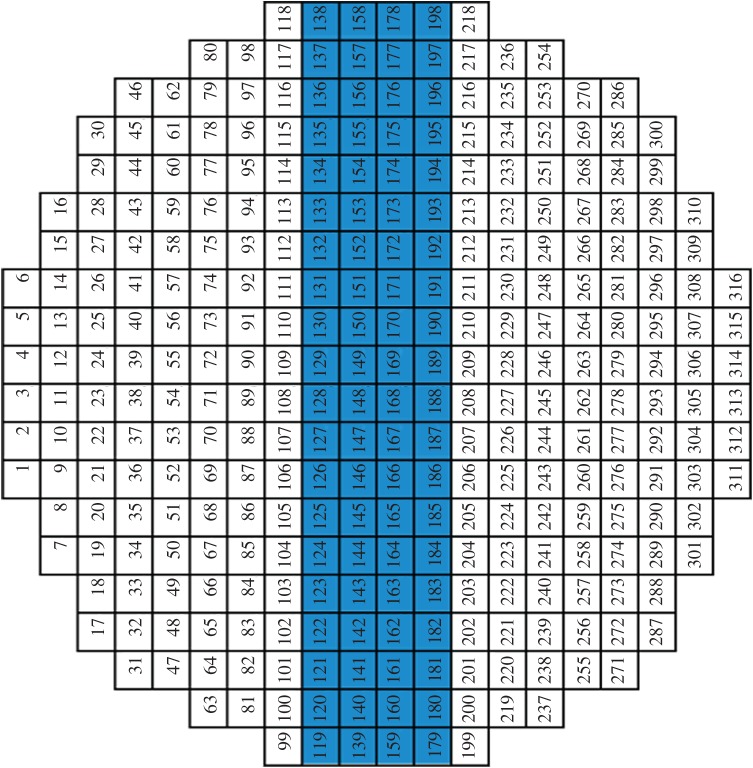
The central columns with 80 elements in the arrangement of 316 tomogram elements by 20×20 grid [29]. (Online version in colour.)

**Figure 6.**
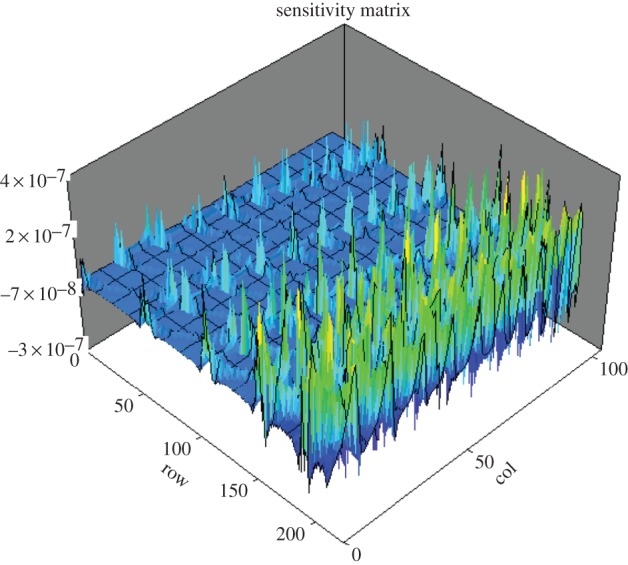
The pixel sensitivity matrix created using FEM with a two-dimensional mesh with 224 triangle pixels for 104 independent measurements from a 16-electrode sensor using adjacent electrode pair sensing strategy. Each row of the matrix contains 104 coefficients, which reports the sensitivity of pixel value in correspondence to measurements.

The characteristics of multi-phase pipeline flow, such as disperse phase concentration and velocity distributions, can be assumed as either radial symmetrical in vertical pipeline or central–vertical plane symmetrical in horizontal pipeline. The necessary and minimum imaging area, which can represent the major features of pipeline flows when both cases are combined, is the columns of pixels along the central–vertical plane, as indicated in figure 5. Therefore, targeting only the central one or few columns of the full tomogram as shown in figure 5 will result in lesser number of measurements thereby increasing the speed while maintaining the spatial resolution to a comparable level. A new sensing strategy, based on 16-electrode EIT, called regional imaging with limited measurements (RILM) was developed in order to achieve similar performance to the conventional EIT sensing protocol but using limited measurements [30]. The selection of measurements can be based on the analysis of sensing regions by either the graphic or numerical simulation as shown in figures 1 and 2, respectively. Alternatively, by analysing the pixel sensitivity matrix, **S**=***τ*A**^T^, the sensitive level of pixel's conductivity owing to the change of boundary measurements can be obtained (figure 6), which provides an analytical method for the implementation of RILM. In the practice, the selection starts with the graphic method would be mostly effective.

**Figure 7.**
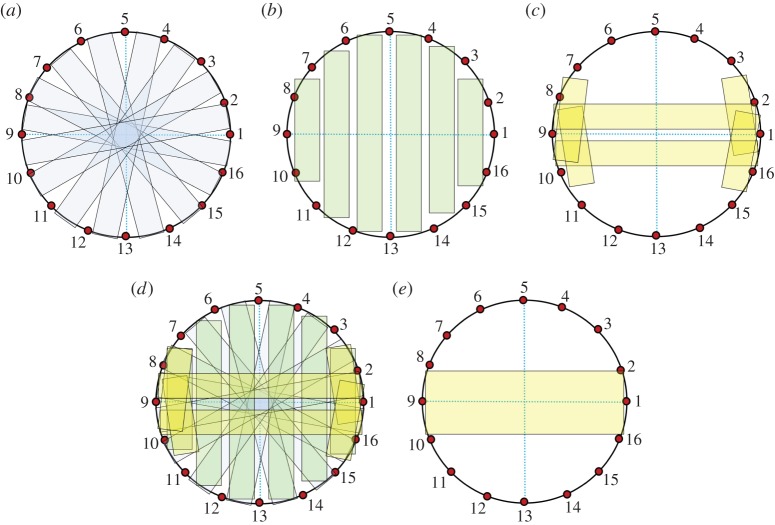
RILM sensing strategy, (*a*) rotary band, (*b*) parallel band and (*c*) complimentary band, (*d*) overall projections and (*e*) region of high sensitivity.

The sensing strategy of the RILM consists of three groups of excitation and measurement electrode pairs arranged in rotary, parallel and complimentary positions as shown in figure 7*a*,*b* and *c*, respectively. Each projection is carried out by applying current to a pair of electrodes and measuring voltage from other pair of electrodes corresponding to the electrode pairs adjacent to the terminals of each rectangular bar in figure 7. A total of 20 measurements, i.e. eight for rotary, six for parallel and seven for complimentary position are required to focus the sensitivity in the central region as illustrated in figure 7*d*,*e* based on the reciprocity theorem.

Further experiments were conducted using the air–water two-phase upward flow in vertical pipeline with inner diameter of 50 mm at the University of Leeds. Slug flow regime was tested with water and air flowing at 27 rpm and 70 litre min^−1^, respectively. A fast 16-electrode EIT system (FICA) was used to collect data at a speed of 1000 dual frames per second. Data for RILM were extracted from the full set of measured data, and velocity and concentration profiles were generated in both cases.

**Figure 8.**
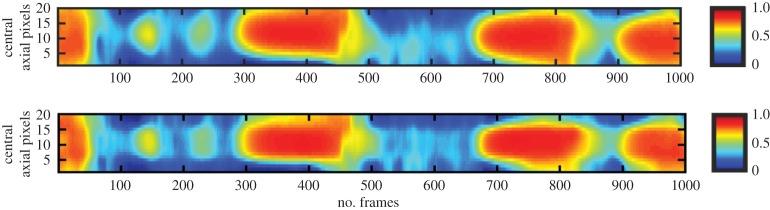
Stacked image of two-phase flow: the top image from full measurement, the bottom image from RILM, where the flowing direction is from the right to left; the red and blue colours in the colour palettes on the right ends of images indicate the volume fraction of air, representing the air and water, respectively.

Stacked images of two-phase slug flow, reconstructed from full measurement, and RILM measurement are shown in figure 8. Average of the central four rows of the tomograms was used in the stacked images. RILM was able to produce comparable visualization with respect to full measurement.

**Figure 9.**
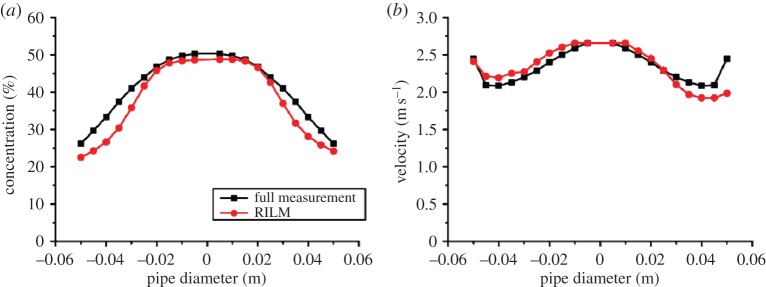
(*a*) Concentration profile, (*b*) velocity profile of air–water vertical flow.

The concentration and velocity profiles were obtained from full measurement as well as RILM for air–water upward two-phase flow in vertical pipeline are illustrated in figure 9*a* and *b*, respectively. The profiles were calculated from the mean value of reconstructed tomograms. Similar characteristics are demonstrated by the profiles obtained from both strategies. The difference between them is more pronounced towards the boundary than the centre of the pipe.

Because RILM requires considerably lesser number of measurements, it is reasonable to anticipate that RILM could outperform conventional EIT systems in terms of measurement speed and communication rate.

## 4. Imaging capability

### (a) Set-ups

**Figure 10.**
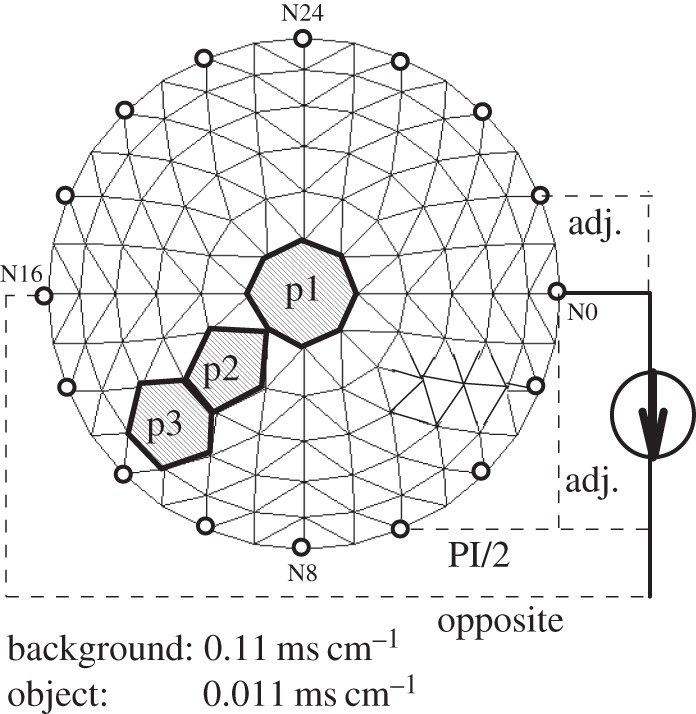
FEM set-ups.

As discussed in §3d, the boundary voltages and the sensitivity matrix for an alternative sensing strategy can be derived from a complete set of independent measurements (equations (3.6) and (3.7)). Therefore, from the algebraic principle, no new information could be obtained from such an arithmetic combination. In practice, the image sensitivity, accuracy and spatial resolution could be affected by the actual signal-to-noise ratio (SNR) and sensitivity distribution of an alternative sensing strategy. To reveal features of the differences, three set-ups of FEM elementary conductivity as defined in figure 10 were employed, in which a low conductive ‘object’ was ‘moved’ from the centre to the side of the domain.

### (b) Imaging capability

**Figure 11.**
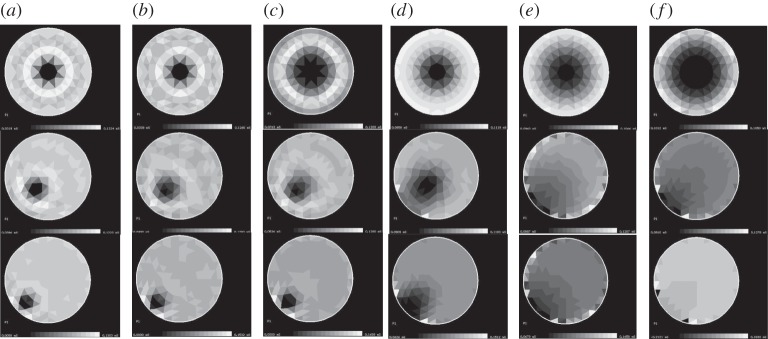
Reconstructed images from the simulated data obtained from adjacent (*a*,*d*), PI/2 (*b*,*e*) and opposite sensing (*c*,*f*) strategies for different conductivity set-ups, p1, p2, p3, as given in figure 9. Images were reconstructed using SCG (*a*–*c*) and SBP methods (*d*–*f*).

Images in figure 11*a*–*c* were reconstructed using the sensitivity theorem-based inverse solution (SCG) [10]. The images obtained from SCG took five iterative steps and 12 iterations for each inverse step. All strategies have a convergent error reduction. The adjacent strategy gives the maximum sensitivity (boundary voltage changes) from these conductivity set-ups and highest convergence speed in the three strategies. The conventional SBP algorithm was also employed to reconstruct the images using the original boundary voltages and sensitivity matrixes of regarded sensing strategy. Significant differences in imaging quality of the three strategies are shown in figure 11*d*–*f*. Comparing the results obtained with SCG algorithm, it is obvious that the difference is due to the diagonal matrix approximation in SBP method. Different imaging qualities have been reached from these results. Images obtained from the adjacent electrode pair strategy have shown the best quality. The poorest quality, particularly at the central region, was delivered from the opposite strategy although the number of measurements (96) in opposite strategy almost closes to the number of measurements from adjacent strategy (104). The images from PI/2 were quite good considering its limited number of measurements (72). It is well known that the quality of resultant image is based on the quantity of acquired information. The quality decay of the image here may be caused by the reduction of information quantity and the ill-conditioned sensitivity matrix, because no noise has been introduced in the simulation. Employing equation (3.4) to analyse the opposite strategy, it can be seen that a large number of repeated basic measurements are involved in the combination. As we discussed in §3d, the applicability of SBP depends on the physical feature of the sensing strategy and whether its diagonal elements of the resolution matrix, ***τ*A**^T^**A**, have the most significant values and therefore can approximate to a diagonal matrix. The imaging quality will highly depend on the feature. Therefore, the SBP is, in general, not applicable to all sensing strategies.

## 5. Imaging of gas–water flows

A considerate set of experiments has been carried out at National Engineering Laboratory in industry-scale experimental flow facilities for the purpose of evaluating electrical tomography technology for new generation of metrology. The pipeline with diameter of 4 inches was horizontally positioned, and the measurement points were located approximately 50 m from the injection points, resulting in fully developed multi-phase flows. Nitrogen, oil with viscosity 19.4×10^−6^ m^2^ s^−1^, and salty water were used as the fluids, with the pressure up to 10 bars. The experiment covered water-cuts with the range of [0%, 100%], combined with gas volume fraction of [0%, 100%], and the flowrate for every phase is 0–140 m^3^ h^−1^. This thorough experiment managed to produce all common flow regimes for horizontal flow, including (wavy) stratified flow, plug flow, slug flow, bubbly flow and annular flow.

**Figure 12.**
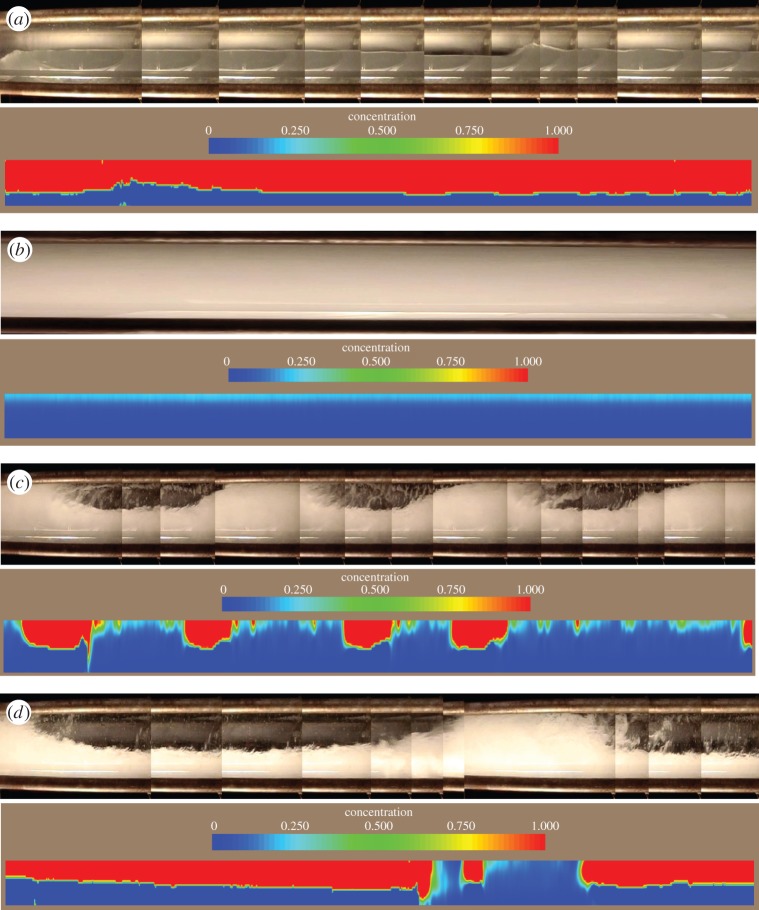
Accumulated axial stacked images of different flow patterns, (*a*) wavy stratified flow (*v*_gas_=39.58 and *v*_liquid_=2.08 l s^−1^), (*b*) bubbly flow (*v*_gas_=2.05 and *v*_*m*_=38.89 l s^−1^), (*c*) plug flow (*v*_gas_=4.17 and *v*_liquid_=23.61 l s^−1^), (*d*) slug flow (*v*_gas_=16.67 and *v*_liquid_= 11.11 l s^−1^). The flow direction is from the right to left.

Accordingly, figure 11 demonstrates only the visualization results for gas–water two-phase flow using voltage-driven EIT system [31]. The flows are visualized with different flow conditions by means of accumulating diameter-direction pixels of cross-sectional concentration tomograms from 2000 frames to form axial cross-sectional stacked images. From flow pattern point of view, figure 11 also indicates different flow regimes, including stratified flow (figure 12*a*), bubbly flow (figure 12*b*), plug flow (figure 12*c*) and slug flow (figure 12*d*).

## 6. Discussion and conclusion

The linear relationship between alternative four-electrode sensing strategies exists. The boundary voltages and the sensitivity matrix for an alternative *four*-electrode sensing strategy can be derived from a complete set of independent measurements. The sensitivity matrix for a specific distribution can also be derived from either an alternative sensing strategy or a particular combination of the sensitivity matrix derived from a known basic set of independent measurements. From algebraic principles, no new information or great accuracy could be obtained from such an arithmetic combination. In practice, the image sensitivity, accuracy and spatial resolution could be affected from the actual SNR and sensitivity distribution achieved from the alternative sensing strategy. The results of the derivation imply that the imaging sensitivity can be *relatively* intensified or attenuated at a particular area of interest by manipulation of matrixes. The applicability of a back-projection algorithm depends on the specific feature of the resolution matrix, ***τ*A**^T^**A**, which is generally determined by the sensing strategy although it can be revised later owing to the linear relationship between four-electrode sensing strategies. Applicability of back-projection (e.g. SBP) is subject to whether its diagonal elements of the transformed matrix, **A**^T^**A**, have the most significant values, and the matrix can approximate to a diagonal matrix. Therefore, the SBP is, in general, not applicable to all sensing strategies. Based on the electrical tomography principle and its sensitivity analysis, electrical tomography imaging may ‘focus’ on part (or sector) of the conventional full image. Based on the method of analysing the pixel sensitive matrix, ***τ*A**^T^, the boundary voltage contribution to each pixel value can be estimated. A specific method, RILM for imaging of a vertical multi-phase flow is demonstrated, which only uses 20 measurements comparing the conventional 104 measurements. Because RILM requires considerably lesser number of measurements, it is reasonable to anticipate that RILM could outperform conventional EIT systems in terms of measurement speed and communication rate.

The clarifications and expressions on the theories of the back-projection method, the linear relationships between sensing strategies and sensitivity matrixes, as well as the RILM have the potential to enhance imaging sensitivity by manipulation of matrixes and to extend electrical tomography to specific applications.
